# Survival of *Clostridioides difficile* spores in thermal and chemo‐thermal laundering processes and influence of the exosporium on their adherence to cotton bed sheets

**DOI:** 10.1111/lam.13811

**Published:** 2022-08-31

**Authors:** J. Tarrant, L. Owen, R. Jenkins, L.J. Smith, K. Laird

**Affiliations:** ^1^ The Infectious Disease Research Group, School of Pharmacy De Montfort University Leicester UK

**Keywords:** adherence, chemo‐thermal disinfection, *Clostridioides difficile*, cotton, detergent, exosporium, hospital, laundry, spores, thermal disinfection

## Abstract

*Clostridioides difficile* spores were previously demonstrated to survive industrial laundering. Understanding interactions between heat, disinfectants and soiling (e.g. bodily fluids) affecting *C. difficile* spore survival could inform the optimization of healthcare laundry processes. Reducing spore attachment to linen could also enhance laundering efficacy. This study aimed to compare the sensitivity of *C. difficile* spores to heat and detergent, with and without soiling and to investigate adherence to cotton. Survival of *C. difficile* spores exposed to industrial laundering temperatures (71–90°C), reference detergent and industrial detergent was quantified with and without soiling. The adherence to cotton after 0 and 24 h air drying was determined with the exosporium of *C. difficile* spores partially or fully removed. *Clostridioides difficile* spores were stable at 71°C for 20 min (≤0·37 log_10_ reduction) while 90°C was sporicidal (3 log_10_ reduction); soiling exerted a protective effect. Industrial detergent was more effective at 71°C compared to 25°C (2·81 vs 0·84 log_10_ reductions), however, specifications for sporicidal activity (>3 log_10_ reduction) were not met. *Clostridioides difficile* spores increasingly adhered to cotton over time, with 49% adherence after 24 h. Removal of the exosporium increased adherence by 19–23% compared to untreated spores. Further understanding of the role of the exosporium in attachment to cotton could enhance spore removal and aid decontamination of linen.

## Graphical abstract

**Significance and Impact of the Study:** *Clostridioides difficile* spores survive on healthcare linens during industrial laundering. This study demonstrates that *C. difficile* spores are resistant to thermal and chemo‐thermal disinfection parameters recommended for healthcare laundry in the UK. The interactions between soiling, heat and disinfectants on *C. difficile* spore inactivation were established, which may aid in optimization of healthcare laundry processes. *Clostridioides difficile* spores attached to cotton over time, with the exosporium playing a role in adherence. These findings may inform novel strategies to prevent attachment to textiles, improving spore removal from linen by detergents during laundering.

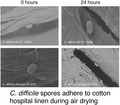

## Introduction

*Clostridioides difficile* is a major cause of healthcare‐associated infections and presents a significant burden in terms of treatment costs, morbidity, and mortality (De Roo and Regenbogen 2020). In England, 12 503 *C. difficile* infections were reported during 2020–2021, including 7109 (57%) healthcare‐associated infections, with the majority of cases in adults over 85 years old, and 1825 deaths within 30 days of infection (UK Health Security Agency 2021). *Clostridioides difficile* produces endospores that are highly resistant to disinfection, desiccation and thermal stress, allowing survival in the environment and mediating transmission of infection (Edwards *et al*. 2016). Symptomatic patients may shed 4–7 log_10_ *C. difficile* spores per gram of faeces (Mulligan *et al*. 1979; Al‐Nassir *et al*. 2008), and therefore *C. difficile* spores are frequently disseminated throughout the ward of infected patients (Gilboa *et al*. 2020). Healthcare environment contamination is considered a risk for *C. difficile* infection, with whole genome sequencing analysis demonstrating nosocomial transmission of genetically related isolates associated with ward contamination (Wen *et al*. 2022).

The United Kingdom healthcare laundry policy, Health Technical Memorandum (HTM) 01–04 (Department of Health 2016), specifies that infectious linen should be thermally disinfected at 65°C for ≥10 min or 71°C for ≥3 min. Chemical disinfection may be employed using alternative times and temperatures only if the decontamination efficacy is equal to or greater than thermal disinfection (Department of Health 2016). According to a survey of 30 UK long‐term care facilities, healthcare linen is most commonly comprised of cotton (50%) followed by polyester‐cotton blend (43·3%; Tarrant 2017). A recent study suggested that *C. difficile* spores can survive on healthcare linen (100% cotton) subject to industrial laundering processes recommended by HTM 01–04 (Tarrant *et al*. 2018). The authors reported that *C. difficile* spores on naturally contaminated cotton bed sheets from *C. difficile* patients, when subject to industrial laundering at 71°C for >3 min with industrial detergent, and with drying and pressing (175°C, 4 bars pressure, 3 s), were only reduced by 0·45 log_10_ CFU 25 cm^−2^ (from 51 to 33 CFU 25 cm^−2^). The HTM 01–04 does not recommend any routine testing of linen for *C. difficile* spores (Department of Health 2016), indicating that current industrial laundering practices could lead to the undetected dissemination of *C. difficile* spores in the healthcare environment (Tarrant *et al*. 2018).

The HTM 01–04 recommended thermal disinfection temperatures were insufficient at inactivating *C. difficile* spores *in vitro*; heating to 71°C for 30 min reduced *C. difficile* spores by 0‐1·30 log_10_ colony forming units (CFU) per ml (Rodriguez‐Palacios *et al*. 2010; Rodriguez‐Palacios and LeJeune 2011). Spores also exhibit resistance to chemicals commonly used within the healthcare environment, such as sodium hypochlorite (Speight *et al*. 2011; Edwards *et al*. 2016), while validated sporicidal agents often require long contact times for sporicidal activity (Horejsh and Kampf 2011; Edwards *et al*. 2016). The presence of soiling (organic matter such as faeces and bodily fluids) may also affect the resistance of spores during laundering, by preventing contact with chemicals and reducing the effects of high temperatures. Artificial soiling significantly reduced the effectiveness of moist heat on *Bacillus subtilis* spores, which survived for significantly longer time periods in the presence of soiling at 94–99°C (Diab‐Elschahawi *et al*. 2010), and reduced the efficacy of chlorine dioxide, triamine and peracetic acid‐based disinfectants against *C. difficile* spores during 60 min of exposure (<3 log_10_ reduction) compared to clean conditions (3 to >4 log_10_ reduction; Edwards *et al*. 2016). The effect of soiling on the survival of *C. difficile* spores during laundering may contribute to thermotolerance, but does not appear to have been reported in the published literature. Further research is required to determine the susceptibility of *C. difficile* spores to temperature and industrial laundering detergents, including the impact of soiling, in order to optimize current laundering parameters.

*Clostridioides difficile* spores adhere to abiotic surfaces, which may enhance their persistence in the environment (Pizarro‐Guajardo *et al*. 2016). There is limited research on the adherence of *C. difficile* spores to textiles such as cotton from healthcare linen; *C. difficile* spores adhered to single‐use spun polypropylene hospital gowns within 10 s of contact (Dyer *et al*. 2019). Inhibition of spore adherence to materials could improve the efficacy of laundering processes as well as the cleaning of hard surfaces by enhancing the removal of spores by detergents. In turn, this would reduce the need for sporicidal agents such as sodium hypochlorite or hydrogen peroxide for textile or surface disinfection. Strategies to prevent attachment could include informing storage parameters of linens prior to laundering, or the development of novel anti‐attachment textile coatings.

There are several factors influencing the adherence of cells and spores to surfaces, such as hydrophobicity, surface roughness and the presence and structure of the exosporial layer (Faille *et al*. 2010; Leishman *et al*. 2010; Joshi *et al*. 2012; Pizarro‐Guajardo *et al*. 2014; Díaz‐González *et al*. 2015). The exosporium is the outermost layer of the spores of several bacterial species; it has been extensively studied in *Bacillus anthracis* and *Bacillus cereus*, but is less well characterized in *C. difficile* (Henriques and Moran 2007; Barra‐Carrasco *et al*. 2013; Pizarro‐Guajardo *et al*. 2014; Díaz‐González *et al*. 2015; Li *et al*. 2016). The exosporium structure varies significantly between isolates of *C. difficile*, ranging from tightly bound to the spore coat with a rough textured appearance to loosely bound and smooth, while some isolates do not possess an exosporium (Malyshev and Baillie 2020). Removal of the exosporium reduces spore hydrophobicity and adherence to Caco‐2 cells (Paredes‐Sabja and Sarker 2012; Escobar‐Cortés *et al*. 2013) and stainless steel (Joshi *et al*. 2012).

The aim of this investigation was to compare the survival of *C. difficile* spores under heat and detergent parameters commonly employed for healthcare laundry and to determine the effects of soiling on the inactivation of *C. difficile* spores. Moreover, this study aimed to quantify the adherence of *C. difficile* spores to 100% cotton and to investigate a possible role of the exosporium in such adherence.

## Results and discussion

In this study, the moist‐heat thermotolerance of *C. difficile* spores, interactions with soiling and laundry chemicals and attachment of spores to cotton were assessed to aid in the potential optimization of industrial laundering.

### Moist‐Heat thermotolerance of *C. difficile* spores and the effect of simulated soiling

**Figure 1:**
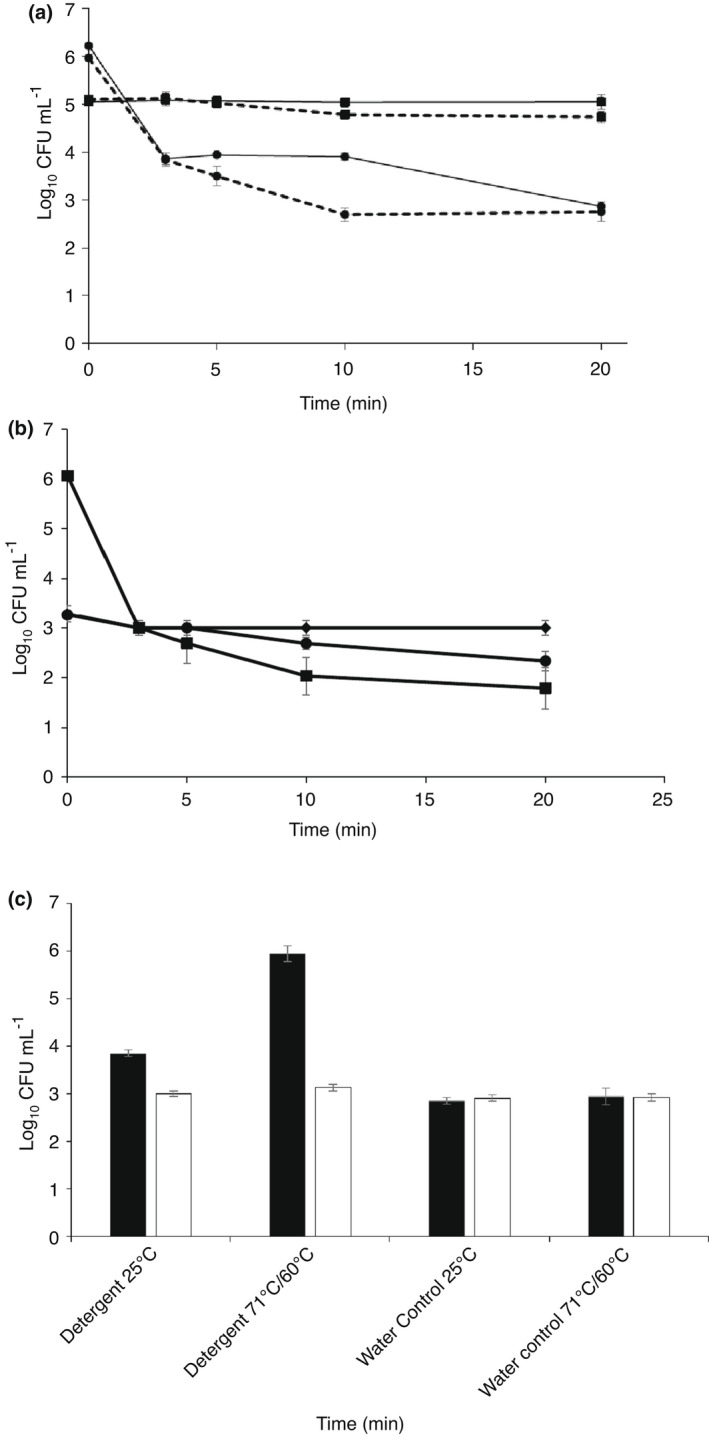
Sensitivity of *Clostridioides difficile* spores to heat and detergents (mean ± SE, *n* = 6). (a) Moist‐heat thermotolerance of *C. difficile* spores at 71°C (■) and 90°C (●) in the presence (solid line) and absence (hatched line) of soiling. (b) Survival in reference detergent at 25°C (♦︎), 71°C (●) and 90°C (■). (C) Survival following treatment with industrial detergent (71°C) followed by bleaching agent (60°C); original inoculum (■) and post‐exposure to test condition (□).

*Clostridioides difficile* exhibited significant resistance to thermal disinfection (Fig. 1a) as previously described (Rodriguez‐Palacios *et al*. 2010). At 71°C for 20 min, the viability of *C. difficile* spores decreased from 5·1 log_10_ CFU per ml to 4·73 log_10_ CFU per ml (*P* ≤ 0·05), in the absence of simulated soiling (3 g L^−1^ bovine serum albumin). In the presence of soiling, there was no reduction in viable spores (5·05 log_10_ CFU per ml), suggesting a protective effect against moist‐heat. The mechanism of this protection is not well understood but could be associated with thermal insulation of spores by the presence of additional proteins (Diab‐Elschahawi *et al*. 2010). The 20‐min exposure time was longer than the thermal disinfection stage of a typical healthcare wash cycle (>3 min + ≥ 8 min mixing time), suggesting that sporicidal and/or surfactant effects of laundry chemistry contribute to the removal and inactivation of *C. difficile* spores from textiles during healthcare laundering (Tarrant *et al*. 2018). The results (Fig. 1a) indicate that *C. difficile* spores were more thermotolerant than previously described; Rodriguez‐Palacios *et al*. (2010) reported a 1·8 log_10_ CFU per ml reduction at 30 min and 2·1 log_10_ CFU per ml reduction after 2 h at 71°C. This study provides further evidence that the recommended HTM 01–04 (Department of Health 2016) thermal disinfection temperature alone is unable to provide complete decontamination. This in turn may represent an infection control risk towards patients using the contaminated linen, and potentially healthcare and laundry workers who handle linen due to self‐contamination during doffing of personal protective equipment (Waterfield *et al*. 2022).

When treated at 90°C, a protective effect from soiling was again evident; the number of recovered spores was significantly (*P* ≤ 0·05) higher in the presence of soiling compared to without soiling after 5 min (3·49 vs 3·94 log_10_ CFU per ml) and 10 min (2·69 vs 3·90 log_10_ CFU per ml, *P* ≤ 0·05; Fig. 1a). In accordance, Diab‐Elschahawi *et al*. (2010) reported that *B. subtilis* spores exhibited a 1 log_10_ CFU per ml reduction at 4 min in the presence of soil, in comparison to 2 min without soiling. In the final 20‐min sample, there was no significant difference (*P* > 0·05) in number of spores recovered whether soiling was absent or present (2·75 log_10_ CFU per ml and 2·86 log_10_ CFU per ml, respectively) and an overall reduction in spore viability of 3·21–3·36 log_10_ CFU per ml was observed (Fig. 1a). By comparison, Rodriguez‐Palacios and LeJeune (2011) reported a >5 log_10_ CFU per ml reduction in viable *C. difficile* spores by 20 min of treatment at 85°C, while Oie *et al*. (2017) reported a >4·98–6·2 log_10_ CFU per ml reductions of *C. difficile* spore type and clinical isolates after 5 min exposure to 90°C. This suggests that isolates of *C. difficile* exhibit differing levels of thermotolerance (Rodriguez‐Palacios *et al*. 2016). A 90°C wash for greater than 10 min may significantly reduce spore contamination in healthcare laundry compared to a 71°C wash, however, the large energy input required to achieve 90°C would be less economical than using sporicidal detergents and additives or facilitating removal of *C. difficile* spores.

### Sporicidal activity of domestic and industrial detergents

The domestic reference detergent was not significantly (*P* > 0·05) sporicidal against *C. difficile* spores over 20 min at 25°C in the presence of soiling (0·27 log_10_ CFU per ml reduction; Fig. 1b). Treatment with domestic reference detergent at 71°C resulted in a small but significant (*P* ≤ 0·05) reduction of 1·43 log_10_ CFU per ml (Fig. 1b), compared to no reduction with temperature alone (Fig. 1a). At 90°C, the domestic reference detergent significant reduced (*P* ≤ 0·05) *C. difficile* spores within 3 min (3·06 log_10_ CFU per ml) with a total reduction of 4·27 log_10_ CFU per ml over 20 min, compared to a 3·36 log_10_ CFU per ml reduction with temperature alone (Fig. 1a).

The combined sporicidal effect of temperature and the industrial detergent (71°C) and bleach additive (60°C) was significant (*p* ≤ 0·05); a 2·81 log_10_ CFU per ml reduction was observed (Fig. 1c). In the absence of heat (25°C), the industrial detergent and bleach additive exposures had a small but significant (*p* ≤ 0·05) sporicidal effect and resulted in a 0·84 log_10_ CFU per ml reduction in spores compared to the water‐only control (Fig. 1c). The reduction of *C. difficile* spores by industrial detergent alone (25°C) was significantly lower than at 71°C, at 0·48 log_10_ CFU per ml, suggesting that the detergent and/or bleach work synergistically with temperature to inactivate spores. Antimicrobials may sensitize spores to a subsequent treatment, such as moist heat (Cortezzo *et al*. 2004). Both domestic and industrial detergents failed to meet the BS EN 13704:2018 sporicidal disinfectant standard threshold of a 3 log_10_ CFU per ml reduction (British Standards Institution, 2018) and were therefore not sporicidal.

### Removal of the *C. difficile* spore exosporium

**Figure 2:**
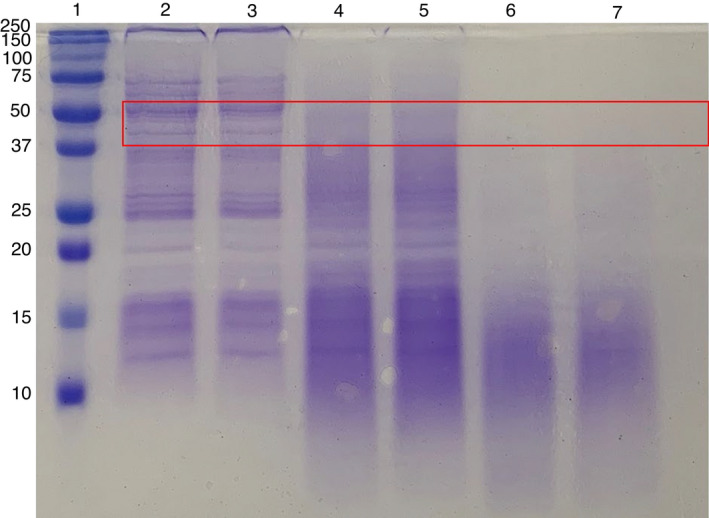
Effect of proteinase K + SDS and trypsin treatments on spore proteins (*n* = 4; representative image shown from duplicate samples). Lane 1, Precision Plus Protein All Blue Protein standards (molecular weights indicated in kDa); Lanes 2 and 3, buffer treated spores; Lane 4 and 5, proteinase K + SDS treated spores; Lanes 6 and 7, trypsin‐treated spores. Loss of protein bands at molecular weights of ~40 to 50 kDa in proteinase K and trypsin‐treated spore samples are highlighted, red box.

The relative hydrophobicity (RH) of spore suspensions was markedly reduced following treatment with trypsin (34·7% RH) and proteinase K + sodium dodecyl sulphate (SDS) (4·1% RH) compared to controls (63·2% RH; Table 1), in line with previous work (Paredes‐Sabja and Sarker 2012; Escobar‐Cortés *et al*. 2013). Proteinase K + SDS treatment had the greatest reduction in hydrophobicity of spores, which is considered indicative of the removal of the exosporium layer, while a smaller reduction in hydrophobicity by trypsin indicates partial degradation of the exosporium (Paredes‐Sabja and Sarker 2012). Denaturing polyacrylamide gel electrophoresis (SDS‐PAGE) of treated spore suspensions showed degradation of proteins at molecular weights of ~40–50 kDa by both trypsin and proteinase K (Fig. 2), which may indicate digestion of the exosporium proteins. This is in accordance with previous research, whereby partial exosporium removal (sonication) released two ~40 to 45 kDa proteins (Paredes‐Sabja and Sarker 2012), while proteinase K treatment decreased the abundance of the *C. difficile* exosporium proteins CotA, CotB, BclA1, BclA2, BclA3, CdeA, CdeB and CdeM which have molecular weights ranging 45–48 kDa (Díaz‐González *et al*. 2015).

**Table 1:** Adherence of *Clostridioides difficile* spores to cotton sheets and effect of exosporial removal on attachment (mean, *n* = 6)

Treatment	Relative adherence to cotton (%)		Log_10_ CFU per ml reduction	RH (%)
	0 h	24 h		
Buffer	10·87	48·71	0·24	63·2
Trypsin	38·34	67·64	0·28	34·7
Proteinase K	66·89	71·82	0·07	4·1

Relative increase in adherence (%) and log_10_ CFU per ml reduction over 24 h after aerobic incubation on NHS 100% cotton sheets and RH of spore samples. Spores were previously treated with buffer (exosporium intact), trypsin (partial removal of exosporium) or proteinase K (full removal of exosporium).

**Figure 3:**
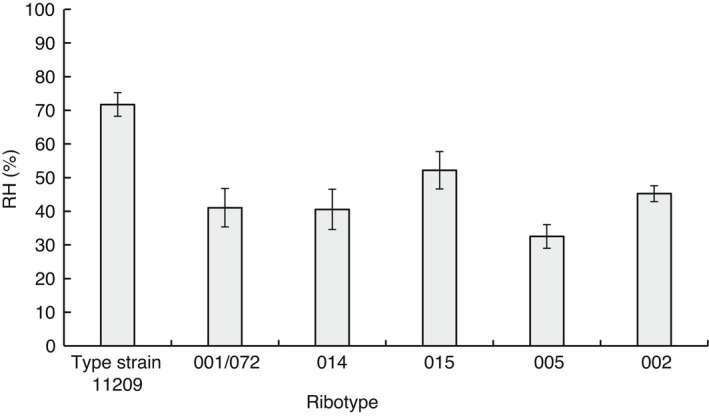
Relative Hydrophobicity (RH; %) of *Clostridioides difficile* NCTC 11209 and clinical isolate spores using the MATH test (mean, ± SE, *n* = 6).

The RH of *C. difficile* NCTC 11209 was significantly greater (*P* ≤ 0·05) than all clinical isolates tested, while the clinical isolates did not significantly differ (*P* > 0·05) in their RH (Fig. 3). RH varied between isolates of the same ribotype, with a significant difference (*P* ≤ 0·05) between *C. difficile* NCTC 11209 (ribotype 001) and clinical isolate ribotype 001/072 (Fig. 3). Substantial variation exists in the exosporium layer between *C. difficile* isolates (Pizarro‐Guajardo *et al*. 2016; Malyshev and Baillie 2020), which may account for variations in RH observed between the type and clinical isolates. Indeed, Joshi *et al*. (2012) reported that the RH of *C. difficile* spore isolates varied between clinical isolates, independent of ribotype (77–14% RH), with an exosporium present for the most hydrophobic isolate (DS1813, 77% RH) but absent from less hydrophobic isolates.

### Adherence of *C. difficile* spores to NHS (100% cotton) sheet swatches

Spores treated with proteinase K + SDS had the highest adherence at 0 and 24 h contact with cotton. However, after an initially lower adherence (0 h, 38·34%) trypsin‐treated spores adhered to a similar extent as those treated with proteinase K + SDS after 24 h (Table 1). There was no significant difference (*P* ≤ 0·05) in the number of untreated spores recovered after 24 h air drying compared to 24 h under anaerobic conditions at room temperature (98·36% vs 98·57% recovery), indicating that air drying did not decrease the viability of the spores. Infected healthcare linen can be stored for up to 24 h before transport to laundering facilities (Tarrant *et al*. 2018), which could allow spores time to adhere and thus potentially reduce the efficacy of laundering; these findings indicate that shorter storage times may be warranted. Further understanding of the parameters involved in spore adherence to linens, such as temperature, humidity and textile composition could inform storage procedures to prevent attachment.

**Figure 4:**
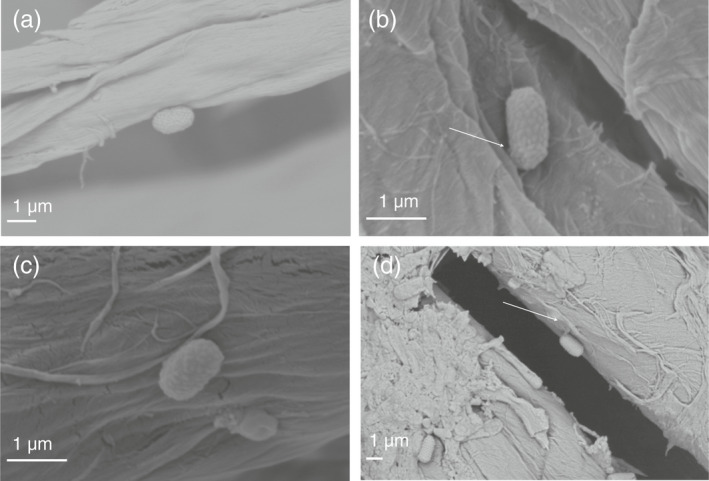
*Clostridioides difficile* spores on 100% cotton swatches. (a) *Clostridioides difficile* NCTC 11209 spore fixed immediately after inoculation, (b) *C. difficile* NCTC 11209 spore fixed after 24 h drying time, (c) *C. difficile* ribotype 001/072 spores fixed immediately after inoculation and (d) *C. difficile* ribotype 001/072 spores fixed after 24 h air‐drying. White arrow = possible anchor structure.

Scanning electron microscope (SEM) images (Fig. 4) further supported the evidence of spores adhering to cotton; the spores remained attached to the surface of the cotton rather than being caught in the weave of the fabric. Possible anchor structures extending from the exosporium to the cotton fibre were evident in an insignificant number of spores for both 0 and 24 h air drying samples (Fig. 4b,d) in accordance with anchor structures observed during spore adherence directly to non‐selective agar plates (Panessa‐Warren *et al*. 1997, 2007). Further investigation would be required to clarify a possible role of this structure in attachment.

The greater adherence of spores with their exosporium removed at 24 h suggests that the exosporium has a negative impact on adherence to cotton. A lower RH, achieved by trypsin and proteinase K treatment, correlated with increased adherence to cotton for *C. difficile* NCTC 11209. Barra‐Carrasco *et al*. (2013) reported that *C. difficile* spores with a defective exosporium layer (Δ*cdeC*) adhered to HT‐29 and caco‐2 intestinal epithelium cells to a greater extent (*P* ≤ 0·05) than wild‐type spores, suggesting that the exosporium layer may decrease initial attachment to cells *in vivo*. In contrast, Paredes‐Sabja and Sarker (2012) reported that removal of the exosporium and hydrophobicity negatively correlated with the attachment of *C. difficile* spores to caco‐2 cells. Paredes‐Sabja and Sarker (2012) and Barra‐Carrasco *et al*. (2013) used different strains of *C. difficile* spores in their studies (630, Pitt51 and Pitt 177 vs R20291) and the method of exosporium removal was different (knock‐out vs chemical/mechanical), which could account for the observed differences in attachment between wild type and exosporium deficient spores. The discrepancy between the current study and that of Paredes‐Sabja and Sarker (2012) may arise from a different mechanism of interaction or attachment of spores to cotton compared to human cells. Further studies into the role of the exosporium in attachment to different surfaces are warranted. This may in turn inform the development of strategies to prevent spore attachment to textiles, such as novel textile coatings/treatments that repel the exosporium.

## Conclusions

This study provides further evidence that *C. difficile* spores are resistant to thermal disinfection temperatures recommended for healthcare laundry in the UK (65–71°C), with ≤0·37 log_10_ reductions after 20 min at 71°C. This suggests that elevated temperatures and/or chemical disinfectants are required for the inactivation of *C. difficile* spores during laundering; indeed, exposure to 90°C for 20 min was sporicidal (>3 log_10_ reduction). Soiling was determined to exert a protective effect against *C. difficile* exposed to heat, further suggesting that laundry chemicals (e.g., surfactants) may be required to improve decontamination efficacy. Increase in temperature improved the efficacy of industrial detergent against *C. difficile* spores, however, the threshold for sporicidal activity (3 log_10_ CFU per ml reduction) was not met. Further optimization of temperature and disinfectant parameters is therefore required. Prevention of spore adherence to textiles is a potential strategy to increase the efficacy of current laundering processes against *C. difficile* spores. This study indicates that *C. difficile* spores attach to cotton over time, suggesting that storage of contaminated linen before laundering could make removal during the wash more difficult. The exosporium may play a role in adherence to cotton. Further discerning the mechanism of adherence could lead to novel strategies to prevent attachment to textiles in the clinical setting.

## Materials and methods

Unless otherwise stated media and chemicals were obtained from Sigma Aldrich, Gillingham, UK.

### Microorganisms

*Clostridioides difficile* NCTC 11209 (ribotype 001) was obtained from the National Collection of Type Cultures (Porton Down, UK). *Clostridioides difficile* infection clinical isolates from ribotypes 002, 005, 014 and 015 were obtained from Public Health England, Newcastle, UK. *Clostridioides difficile* ribotype 001/072 was isolated from soiled hospital bed linen of a *C. difficile* infection patient (Tarrant *et al*. 2018). Ribotyping of clinical isolates was performed by capillary gel electrophoresis, at Public Health England (Newcastle, UK).

### *Clostridioides difficile* spore generation

*Clostridioides difficile* spore suspensions were generated by streaking onto the surface of a brain heart infusion supplemented (BHIS) agar plate: 6% d‐fructose, and 0·1% l‐cysteine and 5% horse blood (TCS, UK). All growth media were pre‐reduced to anaerobic conditions before use. Inoculated plates were incubated anaerobically at 37°C for 48 h. *Clostridioides difficile* colonies were transferred to 20 aliquots of cooked meat broth (10 ml) and incubated anaerobically at 37°C for 14 days. The resulting cultures were pooled before centrifugation at 352 *g* for 20 min. The supernatant was discarded, and pellet resuspended in 5 ml maximum recovery diluent (MRD). The wash was repeated a total of five times to remove cell debris and broth. The final pellet was resuspended in 10 ml MRD and enumerated by spread‐plating (100 *μ*l) on BHIS agar with 0·1% sodium taurocholate acid salt (BHIS/T) in duplicate.

### Moist‐heat thermotolerance of *C. difficile* spores and the effect of simulated soiling

*Clostridioides difficile* NCTC 11209 spores suspended in 1 ml MRD (clean conditions) or 1 ml MRD with 3 g l^−1^ bovine serum albumin (BSA) as simulated soiling were exposed to 71°C (5 log_10_ CFU per ml) or 90°C (6 log_10_ CFU per ml) for 3, 5, 10 and 20 min with 2 *g* shaking. The test suspension was vortexed at the end of the exposure time and spread plated (100 *μ*l) in duplicate on to BHIS/T. The remaining test suspension was membrane filtered (0·45 μm; Whatman, UK) and the membrane was transferred onto BHIS/T for enumeration. Viable *C. difficile* spores were enumerated following anaerobic incubation at 37°C for 48 h. A viability control of the untreated spore suspension was included.

### Sporicidal activity of domestic and industrial detergents

The sporicidal efficacy of domestic ECE non‐phosphate reference detergent A (SDC, Holmfirth, UK) and an industrial detergent (Washing Systems Ltd., Warrington, UK) against *C. difficile* was investigated in the presence of simulated soiling. Domestic detergent testing was conducted at 25, 71 and 90°C. Industrial detergent testing was conducted in two stages: an initial treatment with detergent followed by a treatment with bleach additive, which were conducted at 71 and 60°C, respectively, or at 25°C. Stock solutions of the domestic reference detergent (20·8 g l^−1^ detergent base, 5·4 g l^−1^ sodium perborate and 0·8 g l^−1^ tetraacetylethylenediamine), industrial detergent (0·34% v/v detergent mix part 1, 0·0872% detergent mix part 2 and 0·0872% detergent mix part 3) and industrial bleach additive (0·147% v/v) were prepared in sterile distilled water (SDW).

Neutralization of detergents for viable counting was achieved by membrane filtration using a method adapted from BS EN 13074:2002 (British Standards Institution, 2002). Membrane filters (0·45 *μ*m; Whatman, Little Chalfont, UK) were primed with 50 ml SDW prior to filtration of the test sample, followed by rinsing with 150 ml SDW. The neutralization method was validated by adding 0·1 ml detergent to the primed membrane, rinsing with 150 ml SDW and then filtering 0·1 ml spore suspension (3 log_10_ CFU per ml) suspended in 50 ml. Membrane filters were incubated on BHIS/T and enumerated. There was no significant difference (*P* > 0·05) in viable spores between the inoculum, SDW control and detergent samples (data not shown).

Spores were suspended in MRD and BSA (3 g l^−1^ final test concentration) to yield 3 log_10_ CFU per ml for tests at 25 and 71°C or 6 log_10_ CFU per ml for tests at 90°C. For the reference detergent, an 800 *μ*l aliquot of pre‐warmed reference detergent stock solution was mixed with 200 *μ*l pre‐warmed spore suspension and exposed to 25, 71 or 90°C with 2 *g* shaking for 3, 5, 10 or 20 min. After the defined exposure time, the test suspensions were membrane filtered as described above and surviving spores enumerated. For the industrial detergent, the spore suspension (200 *μ*l) was mixed with pre‐warmed industrial detergent stage 1 stock solution and incubated at 25 or 71°C in a rotating water bath at 2 *g* for 11 min. The mixture was then centrifuged at 3913 *g* for 1 min and the pellet was resuspended in 1 ml freshly prepared, pre‐warmed industrial bleach additive. The test mixture was incubated at 25 or 60°C for 5 min with 2 *g* shaking before membrane filtration and enumeration of surviving spores. A control of spores treated with SDW only was included in all experiments.

### Removal of the exosporium

Spore suspensions were washed with ice‐cold SDW 10 times (1 409 *g*; 5 min; 4°C) and resuspended to 10 ml in SDW. The washed suspension was layered onto 5 ml of 50% Histodenz™ (w/v), centrifuged and the free spore phase resuspended in 10 ml SDW. Histodenz treatment significantly (*P* ≤ 0·05) increased the percentage RH of spores from 3·62% ± 0·98 to 12·08% ± 2·77 using the microbial adhesion to hydrocarbon (MATH) test described below. Purified *C. difficile* NCTC 11209 spores (7 log_10_ colony forming units (CFU) per ml) were incubated in 25 mmol L^−1^ phosphate buffer alone, trypsin (0·15 mg ml^−1^), or proteinase K (0·3 mg ml^−1^) and SDS (10 mg ml^−1^) using a method based on that of Escobar‐Cortés *et al*. (2013). Spores were pelleted and re‐suspended in 30 *μ*l treatment solution, then incubated at 37°C for 2 h with agitation at 8 *g*. The spores were washed five times in SDW, resuspended in 5 ml SDW and enumerated. Treated spore suspensions were stored at −80°C until use. Storage at −80°C did not significantly alter the RH of spores compared to those stored at 4°C (*P* > 0·05) using the MATH test described below (data not shown).

### MATH test of hydrophobicity

The hydrophobicity of the spores of *C. difficile* NCTC 11209 and clinical isolates from ribotypes 001/072, 002, 005, 014 and 015 were assessed using the MATH test, as described by Joshi *et al*. (2012). Spore suspensions were centrifuged at 1409 *g* for 1 min and resuspended in SDW; the optical density at 600 nm (OD_600_) of the spore suspension was then adjusted to 0·5–0·6 using a spectrophotometer (Helios, Thermo Scientific, Newport, UK). A 0·5 ml aliquot of hexadecane (Sigma Aldrich, UK) was added to 2 ml spore suspension, vortexed for 2 min and incubated at room temperature for 15 min. The OD_600_ of the aqueous layer was measured, and the RH (%) of each suspension was calculated as the percentage difference in absorbance before and after incubation in the presence of hexadecane.

### SDS‐page

Trypsin and proteinase K + SDS‐treated *C. difficile* spores were beaten with 0·1 mm silica beads (Lysing Matrix B, MP Biomedicals, Irvine, California, USA) at 6 m s^−1^ using a FastPrep‐24 5G (MP Biomedicals, USA) for 3 × 45 s with 2 min' ice cooling between each interval. The homogenate was centrifuged at 16 000 *g* for 5 min; the supernatant was then diluted with 4× NuPAGE LDS sample buffer (Invitrogen, Waltham, Massachusetts, USA) and boiled (70°C) for 10 min. Controls were spores and supernatants treated with buffer alone. Protein extracts were loaded onto a 15% SDS‐PAGE gel along with a protein size marker (Precision Plus Protein™ All Blue Standard, Bio‐Rad, Hercules, California, USA) and electrophoresed in Tris‐glycine‐SDS buffer at 180 V for approximately 30 min before Coomassie blue staining (Sigma Aldrich).

### Adherence of *C. difficile* spores to cotton

NHS (100% cotton) sheet swatches (25 cm^2^) were inoculated with 0·1 ml of *C. difficile* NCTC 11209 spore suspensions treated with phosphate buffer, trypsin or proteinase K + SDS (5·0–5·5 log_10_ CFU per ml). The spores were recovered immediately, or after 24 h air‐drying, into 30 ml MRD by vortexing five times for 5 s at 40 Hz. The recovered spore suspensions were serially diluted in MRD, duplicate 0·1 ml samples were spread‐plated onto BHIS/T and incubated anaerobically for 48 h at 37°C. Calculations were carried out to assess the difference in relative adherence (RA) % to the cotton fabric, according to the formula:

$$100-\left[\left(\text{Final}\;\mathrm{CFU}\;\mathrm{per}\;\mathrm{ml}/\text{Inoculated}\;\mathrm{CFU}\;\mathrm{per}\;\mathrm{ml}\right)\times 100\right]$$

### Scanning electron microscopy of *C. difficile* spores on cotton

NHS (100% cotton) sheet swatches (25 cm^2^) were inoculated with 0·1 ml of either a *C. difficile* NCTC 11209 or ribotype 001/072 spore suspension (7 log_10_ CFU per ml). The swatches were then fixed immediately or after 24 h drying at room temperature.

Samples of 1 cm^2^ were taken from the centre of the 25 cm^2^ swatches and immersed in 2·5 ml of 2% glutaraldehyde for 4 h to inactivate and fix the spores. The fixative was removed, and the swatch was immersed in 5 ml of phosphate buffer for 10 min. The fixed swatches were dried using an ethanol series of 50, 70, 90 and 100% for 10 min at each concentration, then left for 24 h to air‐dry. Individual swatches were mounted on 2·5 cm aluminium stubs and sputter coated with gold (Edwards Sputter Coater, S150B). The stubs were viewed using an SEM (Carl Zeiss Evo HD 15), in high vacuum with a beam accelerating voltage of 5–7 kV for magnifications between 2500 and 50 000.

### Statistical analysis

All investigations were carried out in triplicate, using independent batches of spores, on at least two separate occasions (*n* = 6).

Statistical analysis was performed with SPSS version 22 (IBM, Portsmouth, UK). Independent *t*‐tests or one‐way analysis of variance with Tukey's *post‐hoc* tests were performed to compare the recovery of *C. difficile* spores between conditions. Where assumptions of normality and homogeneity of variance were violated according to Shapiro–Wilk and Levene's tests, the Mann–Whitney *U* test or Kruskal‐Wallis test with *post‐hoc* multiple comparisons and adjusted significance were performed.

## Conflict of Interest

The authors declare that there is no conflict of interest.

## Author Contributions

Joanna Tarrant: Conceptualization, Methodology, Investigation, Formal analysis and Writing—original draft. Lucy Owen: Investigation, Methodology, Formal analysis, Writing–original draft. Richard Jenkins: Conceptualization, Methodology, Formal analysis, Writing—review and editing. Laura J Smith: Methodology, Formal analysis, Writing—review and editing. Katie Laird: Conceptualization, Methodology, Formal analysis, Writing – review and editing.

## Data Availability

The data that support the findings of this study are available from the corresponding author upon reasonable request.
